# Zebrafish and CRISPR—A synergistic approach to decipher and cure human diseases

**DOI:** 10.1002/ame2.70141

**Published:** 2026-03-23

**Authors:** Manikandan Sivaprakasam, Aashika Raagavi Jeanpierre, Salma Mohammed, Rajesh Srinivasan, Aman Kumar Mohanty

**Affiliations:** ^1^ Mahatma Gandhi Medical Advanced Research Institute (MGMARI) Sri Balaji Vidyapeeth (Deemed to be University) Puducherry India

**Keywords:** animal model, Cas9, CRISPR, *Danio rerio*, genome editing, zebrafish

## Abstract

Rapidly emerging infectious and genetic diseases demand robust vertebrate models to investigate pathogenesis and accelerate therapeutic discovery. Zebrafish (*Danio rerio*) offer substantial translational value owing to their conserved physiology, optical transparency, rapid reproduction, and the presence of orthologs for approximately 70% of human genes and approximately 82% of disease‐associated genes. The integration of CRISPR/Cas9 technology has transformed zebrafish research, enabling efficient generation of targeted knockouts, knockins, and high‐throughput mutagenesis screens. This synergy supports mechanistic dissection and modeling of cardiovascular, oncologic, viral, and other genetic disorders. Despite these advantages, rigorous allele validation, consideration of paralog redundancy, maternal contribution, and off‐target analysis remain essential to ensure translational accuracy. This review summarizes current applications, methodological advances, limitations, and best‐practice recommendations for combining zebrafish models with genome editing to improve understanding and treatment of human diseases.

## Graphical abstract

Zebrafish, with high genetic homology to humans, serves as a powerful vertebrate model for disease modeling and drug discovery. Integration of CRISPR/Cas9 technology enables precise genome editing, facilitating the development of translational models for human diseases. This synergistic approach accelerates insights into pathogenesis and therapeutic strategies.

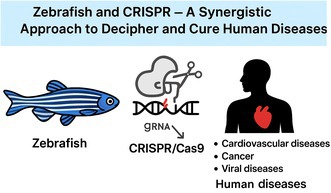

## 1 INTRODUCTION

The zebrafish (*Danio rerio*) is a small tropical freshwater fish found in the rivers and streams of South Asia. Over the past two decades, it has become one of the most powerful vertebrate model organisms in biomedical research. Zebrafish offer a unique combination of advantages—rapid external development, high fecundity, optical transparency during embryogenesis, and genetic tractability—making them particularly suitable for studying vertebrate development, organogenesis, and human disease mechanisms.

Because zebrafish share extensive genomic and physiological similarities with humans, they serve as an exceptional translational model for studying human disorders. In fact, orthologs exist for about 70% of human genes and about 82% of human disease‐associated genes.^1^ These similarities enable functional modeling of numerous human genetic conditions, providing insight into the pathogenesis and progression of disease and facilitating preclinical testing of therapeutic candidates.

The rapid, widespread improvement of genetic tools—particularly CRISPR/Cas9—has transformed zebrafish research, allowing site‐specific genome modification with unprecedented precision and efficiency. This revolution has accelerated the generation of disease models that faithfully recapitulate human phenotypes and has expanded the scope of zebrafish applications from developmental biology to toxicology, cancer, cardiovascular, and infectious disease research.

Compared to traditional mammalian systems such as mice, zebrafish offer multiple experimental and ethical advantages, including smaller size, lower maintenance costs, faster generational turnover, and the ability to perform large‐scale screens. The combination of optical transparency, vertebrate complexity, and efficient genome editing makes zebrafish a uniquely versatile system for mechanistic discovery and translational modeling.

Nevertheless, certain limitations—such as genetic buffering by paralogs, differences in organ physiology, temperature constraints, and innate‐immune dominance during early development—must be carefully considered to ensure valid interpretation of results. Addressing these challenges requires standardized experimental design, robust validation of mutant alleles, and critical comparison with mammalian models.

This review provides an integrated overview of how zebrafish and CRISPR/Cas9 technology complement each other in modeling and potentially treating human diseases. It summarizes the major advantages, experimental workflows, and caveats of the zebrafish system while emphasizing best practices and realistic expectations for translational applications.

## 2 SCOPE AND LITERATURE SELECTION

This narrative review was compiled from systematic searches of PubMed, Google Scholar, and Scopus covering the period till 2025. Studies were included when they reported in vivo zebrafish experiments using at least two independent alleles or a validated rescue, and knockin reports were considered only if germline transmission was demonstrated. We prioritized original research articles describing CRISPR/Cas9‐based genome editing in the context of human disease modeling, focusing on cardiovascular, oncologic, and viral disorders, supplemented with key review and method papers for background. This explicit scope statement clarifies the review's coverage and ensures transparency in literature selection.

### 2.1 Alternative animal models—Latest trend

Model organisms such as mice (*Mus musculus*), fruit flies (*Drosophila melanogaster*), nematodes (*Caenorhabditis elegans*), and yeast (*Saccharomyces cerevisiae*) have long served as indispensable systems for investigating human gene function and disease. Each provides unique advantages—mice for mammalian physiology and immunology, *Drosophila* and *C. elegans* for genetic simplicity and high‐throughput screening, and yeast for cellular and biochemical studies.

However, these models differ in cost, throughput, and physiological relevance. Mice closely resemble humans but are expensive and slower to breed; invertebrates allow rapid and inexpensive experimentation but lack key vertebrate organs and immune components. Zebrafish bridge this gap by combining vertebrate complexity with high fecundity, transparent embryos, and ease of genome manipulation.

Their small size, external fertilization, and short‐generation time enable genetic studies at a scale and speed unattainable in mammals while maintaining organ systems, including cardiovascular, nervous, hematopoietic, and immune networks, comparable to humans. As such, zebrafish occupy a critical middle ground between invertebrate simplicity and mammalian physiological fidelity.

**FIGURE 1:**
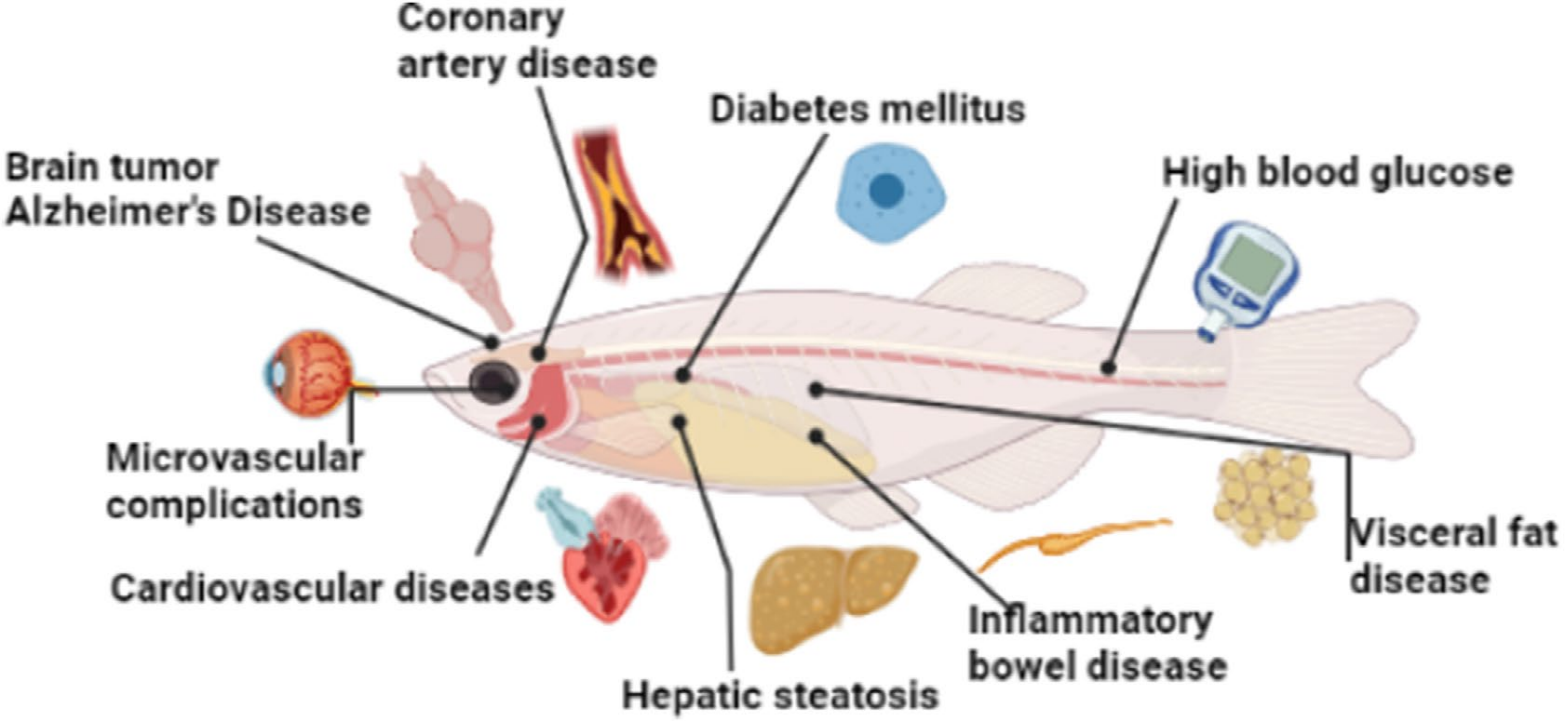
Anatomical similarities between zebrafish (*Danio rerio*) and higher vertebrates. These comparative schematic highlights conserved organ systems shared between zebrafish and mammals, including the brain, heart, liver, kidney, and gastrointestinal tract. These anatomical parallels make zebrafish a robust translational model for studying vertebrate organ development, physiology, and pathology. Figure created with BioRender.com; minor adjustments made for clarity.

This balance makes zebrafish a practical in vivo vertebrate model for translational discovery, capable of complementing and validating results from both mammalian and invertebrate systems. When integrated with powerful genome‐editing technologies such as CRISPR/Cas9, zebrafish emerge as one of the most versatile platforms for modeling and dissecting human diseases (Figure 1).

### 2.2 Zebrafish as a unique animal model

The zebrafish (*D. rerio*) is a small tropical freshwater fish found in the rivers and ponds of South and Southeast Asia. Owing to its external fertilization, transparent embryos, and rapid development, zebrafish enable direct observation of developmental processes from the earliest stages. Embryogenesis completes within 72 h postfertilization, allowing rapid generation and phenotypic screening of mutants.

Zebrafish are easy to maintain, require minimal space, and produce large numbers of offspring, making them highly cost‐effective compared to mammalian models such as mice. Their short‐generation time (approximately 3 months to sexual maturity) supports efficient breeding and multigenerational studies.

These features have led to the rapid, widespread adoption of zebrafish in developmental biology, genetics, and disease modeling laboratories worldwide. The species' optical transparency, coupled with advanced live‐imaging technologies, permits real‐time visualization of organ formation, neuronal activity, and disease progression in living animals.

**FIGURE 2:**
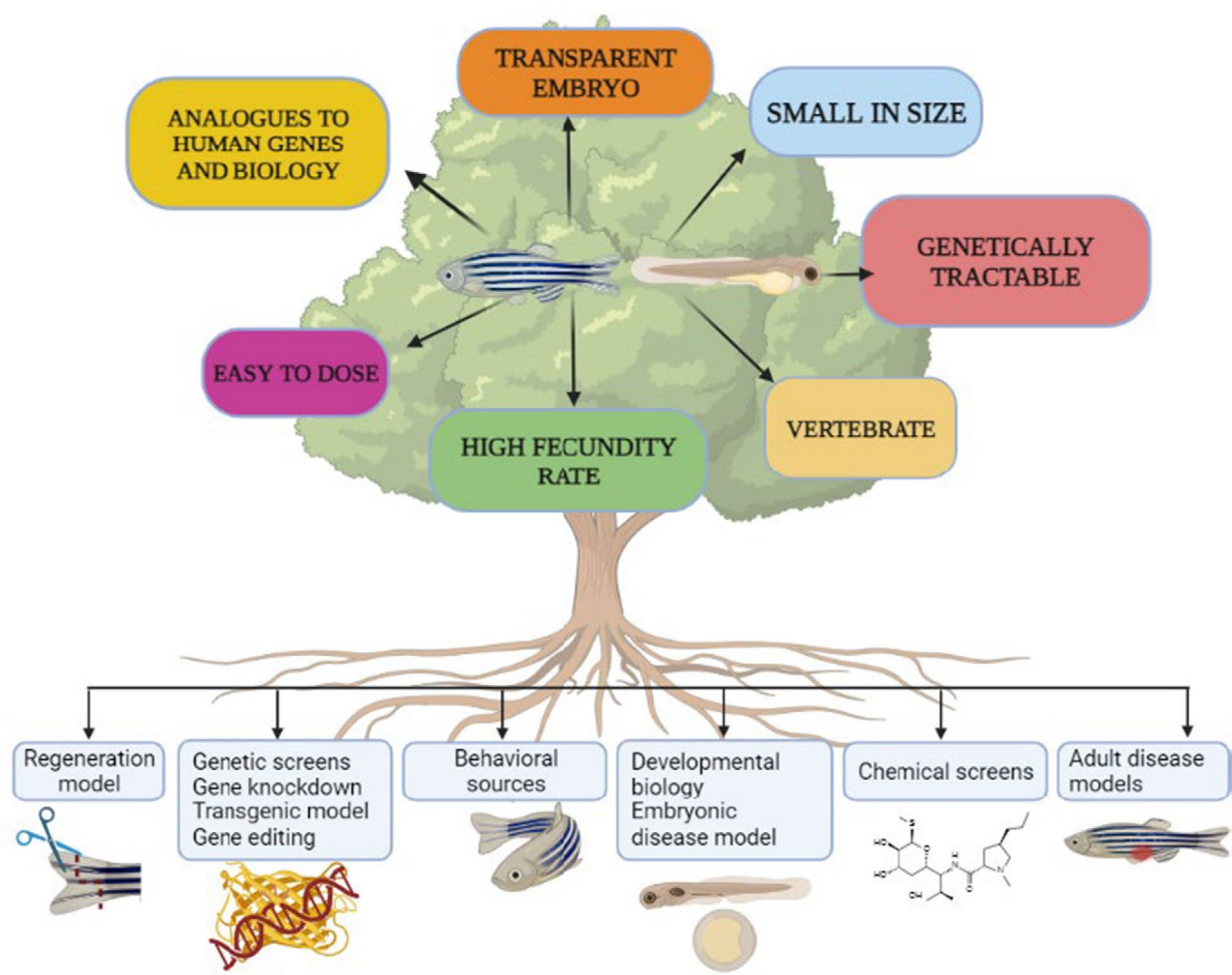
Zebrafish (*Danio rerio*) as a unique vertebrate model organism for biomedical research. This figure summarizes the key experimental advantages of zebrafish: Optical transparency during early development, high fecundity, rapid organogenesis, external fertilization, and genetic similarity to humans—about 70% of human genes and 82% of human disease‐associated genes have zebrafish orthologs.^1^ These features make zebrafish highly suitable for in vivo imaging, gene‐editing studies, and drug screening. Figure created with BioRender.com; minor adjustments made for clarity.

Furthermore, the availability of numerous transgenic and mutant lines, together with powerful genome‐editing tools such as CRISPR/Cas9, has firmly established zebrafish as an indispensable vertebrate model for investigating gene function and human pathology (Figure 2).

### 2.3 Metabolism and immune system of zebrafish

Zebrafish exhibit metabolic and immune system organization broadly comparable to that of higher vertebrates, making them suitable for modeling human physiological and pathological processes. The species possesses functional analogs of most mammalian organs, including the liver, kidney, pancreas, and hematopoietic tissues, allowing direct assessment of metabolic, endocrine, and detoxification pathways.

At 28–29°C, the standard rearing temperature for zebrafish, enzymatic and metabolic activities are temperature‐adapted but remain sufficiently conserved to permit meaningful comparison with mammalian systems. However, pharmacokinetic parameters such as absorption, distribution, and clearance must be interpreted cautiously, because compounds are often administered via water or feed rather than systemic injection.

The zebrafish immune system develops progressively, with innate immunity predominating in embryos and larvae and adaptive immunity maturing around 4 to 6 weeks postfertilization. The innate immune components—macrophages, neutrophils, and complement proteins—are fully functional within 48 h postfertilization, enabling early modeling of host–pathogen interactions and inflammatory processes. Adaptive immunity, mediated by functional B and T lymphocytes, emerges later and parallels the mammalian organization of thymus, spleen, and kidney marrow.

This temporal separation between innate and adaptive responses is particularly advantageous for dissecting early inflammatory signaling and evaluating innate‐targeted therapies. Nevertheless, researchers should recognize that larval studies predominantly reflect innate immunity, and results must be confirmed in juvenile or adult fish when adaptive mechanisms are relevant.

### 2.4 The making of an interesting translational model

Zebrafish have become a cornerstone of translational research because their genetic, physiological, and developmental characteristics align closely with those of humans. Approximately 70% of human genes and 82% of human disease‐associated genes have identifiable zebrafish orthologs,^1^ allowing functional modeling of numerous disorders at the organismal level. Such conservation makes zebrafish an exceptional system for exploring gene–phenotype relationships that are directly relevant to human biology.

The transparency of zebrafish embryos enables continuous, noninvasive imaging of developing organs, tumors, and regenerative processes, offering experimental access unmatched in mammalian models. Combined with powerful transgenic lines expressing fluorescent reporters in specific tissues, researchers can visualize dynamic processes such as angiogenesis, neurogenesis, immune cell trafficking, and tumor invasion in real time.

Physiologically, zebrafish share many fundamental systems with mammals, including a closed circulatory system, conserved neurotransmitter pathways, and homologous endocrine regulation, providing a realistic platform for studying cardiovascular, neurological, metabolic, and developmental disorders.

The integration of CRISPR/Cas9 genome editing with these intrinsic advantages now allows rapid generation of targeted mutants and humanized lines, transforming zebrafish into a highly tractable vertebrate model for precision medicine. This synergy bridges basic biology with translational research, facilitating preclinical screening of drugs, validation of therapeutic targets, and mechanistic exploration of human diseases in a living vertebrate organism.

In summary, the zebrafish combines experimental accessibility with vertebrate complexity, offering a balanced compromise between in vitro cellular systems and costly mammalian models. These attributes make it an exceptionally powerful translational model for investigating the genetic and molecular foundations of human health and disease.

### 2.5 Genetic buffering and maternal contribution

Genetic buffering and maternal contribution represent two key biological phenomena that can obscure or modify phenotypes in zebrafish gene‐editing experiments. Understanding these effects is essential for accurate data interpretation and for designing robust validation strategies.

#### 2.5.1 Paralog and ohnolog redundancy

Following the teleost‐specific whole‐genome duplication, many zebrafish genes exist as duplicate pairs known as ohnologs, which can compensate for one another and mask mutant phenotypes.^2^ Such genetic redundancy often conceals the consequences of single‐gene knockouts, necessitating double‐mutant or paralog‐specific analyses to uncover true gene function. Researchers should therefore examine the expression of both paralogs and, when necessary, generate compound or double mutants to test for functional overlap.

#### 2.5.2 Transcriptional adaptation

Beyond gene duplication, zebrafish exhibit a compensatory mechanism called transcriptional adaptation, where mutant messenger RNA (mRNA) decay triggers upregulation of related genes.^3^, ^4^ This response can blunt or completely rescue phenotypes in CRISPR/Cas9 knockouts, creating apparent false‐negative results. To minimize misinterpretation, investigators should compare two independent alleles (for instance, a small frameshift and a larger domain‐deleting allele), confirm loss of transcript or protein, and perform phenotypic rescue experiments where feasible.

#### 2.5.3 Maternal contribution and developmental masking

During early development, maternally deposited mRNAs and proteins sustain embryogenesis until the maternal‐to‐zygotic transition (MZT). Consequently, zygotic mutants for early‐acting genes may appear phenotypically normal because of maternal product persistence.^3^ To reveal these masked roles, maternal–zygotic (MZ) mutants or maternal knockdowns should be generated. When early lethality or developmental arrest is suspected, researchers should examine maternal, zygotic, and MZ contexts to distinguish stage‐specific effects.^5^

Best‐practice recommendations are described below:

1. Assess paralog expression for candidate genes likely to have ohnolog.^2^
2. Generate and compare at least two independent alleles.
3. Validate functional specificity through mRNA rescue.
4. Analyze maternal, zygotic, and MZ mutants for early‐expressed genes.
5. Interpret absent phenotypes cautiously, considering buffering and adaptation.

Accounting for ohnolog redundancy, transcriptional adaptation, and maternal effects greatly improves the reproducibility and translational reliability of zebrafish genome‐editing studies.

### 2.6 Genome editing in zebrafish

The advent of CRISPR/Cas9 technology has revolutionized zebrafish genetics, enabling rapid, efficient, and precise modification of the genome.^6^, ^7^ This versatile system allows for targeted knockouts, knockins, large deletions, and site‐specific insertions with a simplicity unmatched by previous methods such as TALENs or ZFNs.

CRISPR/Cas9 genome editing in zebrafish is typically performed by microinjecting Cas9–sgRNA complexes into one‐cell embryos, where the nuclease introduces a double‐stranded DNA break at the target locus. Repair occurs primarily via error‐prone nonhomologous end joining (NHEJ), creating insertions or deletions (indels) that disrupt gene function. For knockin applications homology‐directed repair (HDR) or short‐homology strategies such as homology‐mediated end joining (HMEJ) or GeneWeld can introduce desired sequences with greater efficiency.^8^, ^9^

Preassembled ribonucleoprotein (RNP) complexes, comprising purified Cas9 protein and synthetic single‐guide RNA (sgRNA), have become the standard and most efficient delivery format.^10^, ^11^ Compared to plasmid or mRNA injections, RNPs reduce off‐target effects and ensure transient nuclease activity, thereby minimizing unintended mutagenesis. Alternative delivery methods, such as Cas9 mRNA and sgRNA coinjection, remain useful for large‐scale or multiplexed functional screens.^12^

**FIGURE 3:**
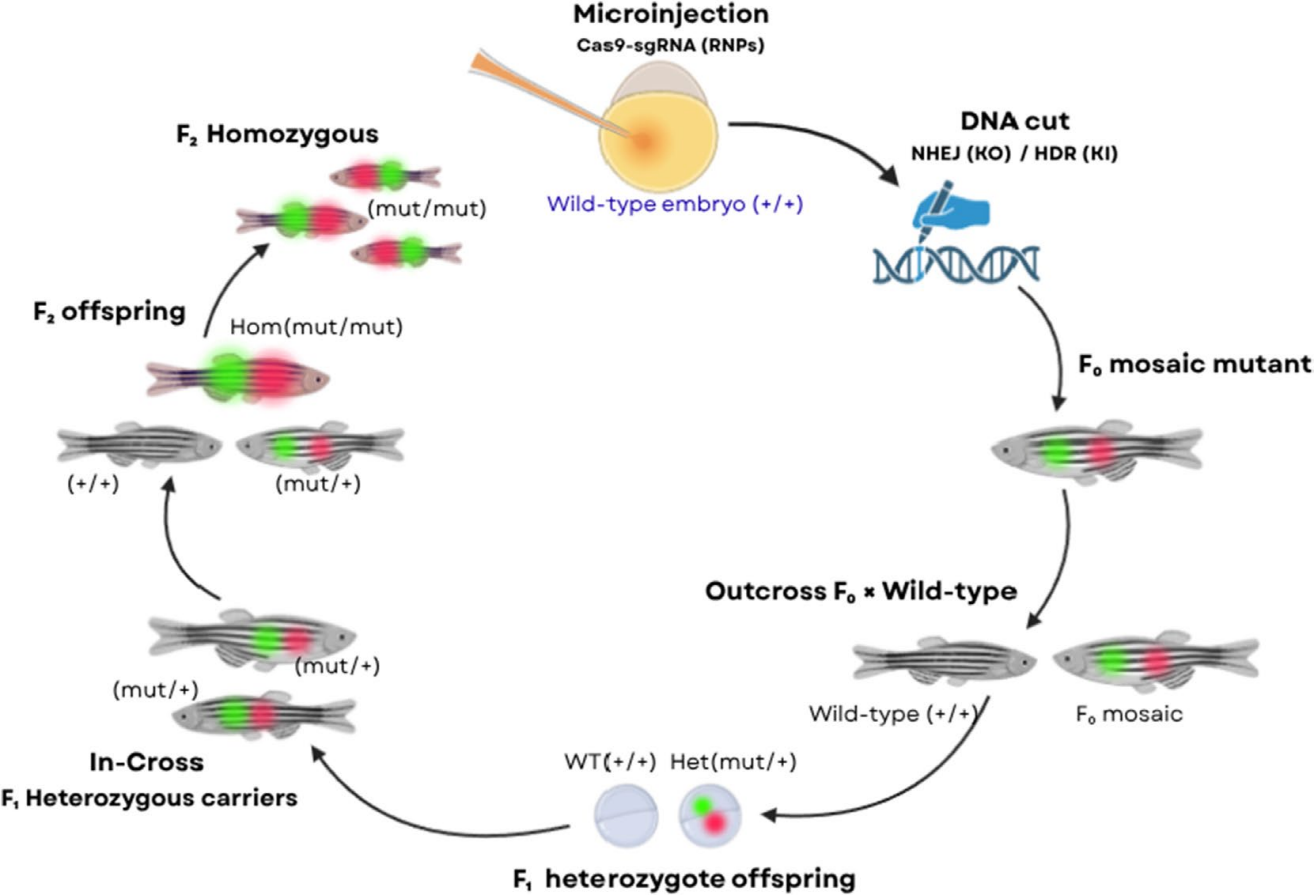
One commonly used CRISPR/Cas9 workflow in zebrafish. The workflow illustrates injection of Cas9‐sgRNA ribonucleoproteins (RNPs) or Cas9 mRNA/sgRNA into one‐cell embryos, induction of DNA double‐strand breaks, and repair by nonhomologous end joining (NHEJ) or homology‐directed repair (HDR). The generational progression from F_0_ mosaic “crispants” to F_1_ heterozygotes and F_2_ homozygotes is depicted. RNP delivery provides high efficiency with reduced off‐target effects, whereas knockin efficiency can be improved via homology‐mediated end joining (HMEJ) or GeneWeld strategies.^9^, ^14^ WT, wild type; Het, heterozygous; Hom; homozygous; mut, mutant. Figure created with BioRender.com; minor adjustments made for clarity.

One commonly used CRISPR/Cas9 workflow in zebrafish follows a generational progression from mosaic F0 “crispants” to stable F1 heterozygotes and F2 homozygotes^13^ (Figure 3).

- *F0 crispants* are mosaics carrying various indels and serve as valuable tools for rapid phenotype discovery and early functional screening.
- *F1 heterozygotes* confirm germline transmission and provide defined alleles for heritable studies.
- *F2 homozygotes* enable definitive genotype–phenotype correlation and are essential for translational modeling and mechanistic validation.^15^

Off‐target activity in zebrafish depends on sgRNA sequence quality, genomic context, and the nuclease variant used.^16^ Therefore, broad claims of “very low off‐target effects” have been revised to reflect this dependence. Researchers should perform targeted validation of the top three to five predicted off‐target loci by sequencing or restriction‐fragment analysis to ensure precision.

Knockin validation requires sequencing of integration junctions and confirmation of germline transmission, as HDR rates remain modest in zebrafish. Combining CRISPR/Cas9 with optimized donor templates and selection markers has improved efficiency, but precise genome editing remains gene‐dependent and technically demanding.^11^

The synergy between zebrafish and CRISPR/Cas9 has already yielded transformative insights across disease areas, including cardiovascular, oncologic, and infectious disorders.^13^, ^15^ Mutant zebrafish lines now model congenital heart defects, hematologic syndromes, tumor progression, and immune–pathogen interactions with high fidelity. Such advances underscore the zebrafish's value for both discovery biology and translational therapeutic screening.

### 2.7 Caveats and validation considerations

Although CRISPR/Cas9 has revolutionized zebrafish genetics, several experimental and biological caveats must be carefully managed to ensure reliable and reproducible results. Zebrafish embryos exhibit high tolerance to genome manipulation, yet mosaicism, allele variability, and off‐target activity can complicate data interpretation if not properly addressed.

1. F_0_ mosaicism and generational validation: Genome editing at the one‐cell stage often produces mosaic F_0_ “crispants” carrying a mixture of alleles within individual tissues. Such F_0_ fish are valuable for rapid phenotype discovery, but definitive genotype–phenotype conclusions require germline‐transmitted alleles. Therefore, F_0_ → F_1_ → F_2_ progression should be followed whenever stable inheritance is essential.
2. Realistic knockin strategies: HDR is relatively inefficient in zebrafish. More effective methods rely on short‐homology–based approaches, such as HMEJ or GeneWeld, which improve precise insertions and facilitate reporter or tag integration.^8^, ^9^ Knockin validation must include sequencing across integration junctions and confirmation of germline transmission.
3. Allele design and verification: To confirm specificity and exclude artifacts, it is recommended to generate at least two independent alleles per target gene—for example, a small frameshift and a larger deletion removing critical functional domains. Allele structure, germline transmission, and zygosity should be reported explicitly in publications.
4. Off‐target analysis: Although CRISPR/Cas9 is generally accurate, off‐target effects depend on guide sequence, genomic context, and nuclease variant. Researchers should validate the top three to five predicted off‐target loci using polymerase chain reaction (PCR) and sequencing or restriction‐enzyme assays. Use of preassembled Cas9–sgRNA RNP complexes minimizes persistent nuclease activity and lowers unintended mutagenesis.
5. Functional rescue and reproducibility: A definitive demonstration of causality requires phenotypic rescue by expressing the wild‐type gene, preferably both the zebrafish and human orthologs. Such rescue tests confirm that observed defects result from specific gene disruption rather than background variation or technical artifacts.

### 2.8 Zebrafish models in cardiovascular research

Cardiovascular diseases (CVDs) remain the leading cause of mortality worldwide, accounting for an estimated 17.9 million deaths annually.^17^ Understanding the genetic and molecular underpinnings of cardiac development and pathology is therefore essential for improving prevention and therapy. Zebrafish (*D. rerio*) have become an indispensable vertebrate model for cardiovascular biology due to their optical transparency, rapid embryonic development, and genetic tractability.

The zebrafish heart shares striking structural and functional conservation with the human heart, including similar molecular pathways regulating cardiac morphogenesis, contractility, and conduction. Although the zebrafish possesses a two‐chambered heart (one atrium and one ventricle) compared to the human four‐chambered heart, the underlying signaling networks—Notch, *Wnt/β*‐catenin, BMP, and FGF pathways—are evolutionarily conserved.

CRISPR/Cas9‐mediated genome editing has enabled the creation of targeted zebrafish mutants to model human cardiovascular disorders with high precision. For example, disruption of *heg1* (heart of glass homolog 1) using CRISPR/Cas9 produced zebrafish with defective endocardial–myocardial interactions, recapitulating aspects of human congenital heart disease. The *heg1Δ25* mutant displays abnormal heart looping and chamber dilation, demonstrating the gene's essential role in maintaining cardiac integrity.^18^

Similarly, CRISPR/Cas9 knockouts of cardiac transcription factors such as *nkx2.5*, *tbx5*, and *gata4* have yielded phenotypes consistent with human congenital heart defects, validating zebrafish as a relevant in vivo model.^19^, ^20^ Knockin models have also been developed to study arrhythmogenic cardiomyopathies and hypertrophic cardiomyopathy by introducing specific human disease mutations (e.g., *tnnt2a*, *actc1b*, and *myh6* alleles).

Live imaging of fluorescently labeled transgenic lines (such as *Tg(flk1:EGFP)* and *Tg(myl7: DsRed)*) allows detailed visualization of heart morphology, angiogenesis, and blood flow dynamics. Combined with high‐speed confocal microscopy and functional assays (e.g., fractional shortening and heart rate quantification), zebrafish provide unparalleled insights into cardiac physiology and the effects of genome editing on developmental processes.

Pharmacological screening using zebrafish embryos has accelerated cardiovascular drug discovery. Exposure to candidate compounds in transparent embryos allows real‐time monitoring of vascular remodeling, heart rate, and blood pressure phenotypes. CRISPR/Cas9‐based mutants can serve as disease models for preclinical screening, linking genotype to therapeutic response.

Despite their advantages, zebrafish models have certain limitations in cardiovascular research.

1. The two‐chambered heart restricts direct comparison to human cardiac hemodynamics.
2. Zebrafish regenerate cardiac tissue efficiently, unlike mammals, which may alter postinjury responses.
3. Standard zebrafish husbandry occurs at 28–29°C; however, in larval xenograft and patient‐derived xenograft (zPDX) assays, fish are routinely incubated at elevated temperatures (~32–34°C, and in some cases briefly up to ~36°C) to support human tumor‐cell proliferation and invasion while maintaining acceptable larval viability.^21^ This temperature shift influences metabolic, proliferative, and pharmacological responses and must be considered when interpreting translational outcomes.

Nevertheless, the zebrafish–CRISPR/Cas9 platform provides a high‐throughput, genetically tractable, and cost‐effective system for understanding cardiovascular genetics and identifying novel therapeutic targets.^15^

### 2.9 Role in cancer biology

Cancer remains one of the leading global causes of death, imposing a major public health and socioeconomic burden.^22^ The disease is driven by well‐defined *hallmarks of cancer*, including sustained proliferative signaling, evasion of apoptosis, metabolic reprogramming, and immune escape. To study these complex processes and develop new therapeutic approaches, researchers depend on robust vertebrate models that accurately recapitulate tumor biology.

Among such systems, zebrafish (*D. rerio*) have become a powerful model for cancer biology and preclinical research. They offer unique advantages—optical transparency, genetic tractability, and rapid development—that enable in vivo imaging of tumor initiation, angiogenesis, and metastasis. Zebrafish have thus complemented mouse models in pharmacological evaluation, biomarker discovery, and genetic subtyping of tumors.^23^, ^24^, ^25^

### 2.10 CRISPR/Cas9 and oncogene modeling

The CRISPR/Cas9 genome‐editing system has transformed zebrafish oncology by allowing the precise manipulation of oncogenes and tumor suppressor genes. Compared to traditional mutagenesis or transgenesis, CRISPR/Cas9 enables targeted knockouts, knockins, and multiplexed gene modifications with high efficiency and reproducibility.^15^

Somatic or germline editing of cancer‐related genes—such as *tp53*, *braf*, *apc*, and *myc*—has yielded zebrafish models that faithfully mirror human tumorigenesis (Table 1).

- *tp53* mutants display spontaneous tumor formation resembling Li‐Fraumeni‐like syndromes.
- *brafV600E* knockins rapidly induce melanoma within weeks, providing a tractable system for MAPK pathway inhibitor testing.
- *apc* null mutants exhibit intestinal hyperplasia and adenoma formation, mimicking colorectal cancer progression.

**TABLE 1:** Representative zebrafish–CRISPR/Cas9‐based cancer models.

Gene/pathway	Cancer type	Editing strategy	Phenotypic outcome/application	References
*tp53*	Sarcoma, leukemia	CRISPR/Cas9 knockout	Recapitulates spontaneous tumorigenesis resembling Li‐Fraumeni syndrome	[24]
*brafV600E*	Melanoma	Knockin via HDR	Induces rapid melanoma; used for pathway inhibitor screening	[25]
*apc*	Colorectal adenoma	Germline knockout	Promotes intestinal hyperplasia; useful for Wnt‐pathway drug testing	[13]
*myc*	Hepatocellular carcinoma	Conditional CRISPR activation	Drives hepatic tumorigenesis; enables chemical screen for antiproliferative drugs	[15]

Abbreviation: HDR, homology‐directed repair.

Tissue‐specific CRISPR/Cas9 activation using promoters such as *mitfa*, *gata1*, or *fabp10a* allows the creation of organ‐targeted mosaic cancers, supporting mechanistic studies of oncogenic pathways and tumor microenvironment interactions.^24^, ^25^

### 2.11 Imaging and drug discovery applications

Zebrafish embryos and larvae are transparent, enabling high‐resolution live imaging of cancer processes. Transgenic reporters like *Tg(kdrl:EGFP)* and *Tg(mitfa:GFP)* visualize angiogenesis, invasion, and immune cell infiltration in real time. Patient‐derived xenografts (zPDX) allow direct implantation of human tumor cells into zebrafish embryos, offering a fast, cost‐effective preclinical platform for evaluating therapeutic efficacy within 3 to 5 days.

These features make zebrafish particularly valuable for:

- *Mechanistic dissection* of oncogene and tumor suppressor functions.
- In vivo *imaging* of metastasis and angiogenesis.
- *High‐throughput chemical screening* for antitumor agents.

### 2.12 Limitations of zebrafish cancer models

Despite these strengths, zebrafish oncology has intrinsic biological and technical limitations that must be clearly recognized for translational interpretation:

1. Temperature difference: Zebrafish are maintained at 28–29°C, whereas mammalian tumor cells proliferate best near 37°C, influencing metabolic and pharmacological responses.
2. Immune system context: Larval zebrafish rely predominantly on innate immunity; adaptive immune components mature later. Adult xenografts require immunosuppression or immune‐deficient strains, altering host–tumor interactions.
3. Anatomical divergence: Organ analogs (e.g., liver, pancreas, brain) differ structurally, which may affect metastatic behavior and stromal signaling.
4. Pharmacokinetic variability: Drug absorption through water exposure yields uncertain internal concentrations; quantification using liquid chromatography–mass spectrometry (LC–MS) or fluorescence is recommended.
5. Genetic caveats: Mosaicism, transcriptional adaptation, and paralog redundancy may confound CRISPR/Cas9 phenotypes; using multiple alleles and rescue experiments improves reliability.^3^, ^4^

Table 2 summarizes key experimental, genetic, and methodological considerations for the use of zebrafish models in cancer research, including tumor initiation, progression, and translational relevance.

**TABLE 2:** Best‐practice considerations for zebrafish cancer studies.

Aspect	Recommended practice
Genetic design	Use ≥2 independent sgRNAs; validate indels and knockins by sequencing
Phenotype validation	Employ ≥2 alleles (frameshift + deletion); include rescue where feasible
Immunological context	Choose larval (innate) or adult (adaptive) models according to the aim of the study
Drug testing	Quantify exposure and internal concentration before efficacy conclusions
Translation	Confirm zebrafish findings in mammalian or human cell models

### 2.13 Role in viral diseases

Viral infections remain among the most serious threats to global health, requiring experimental models that can reveal host–pathogen interactions and support antiviral drug discovery. Zebrafish (*D. rerio*) have recently emerged as an informative vertebrate model for visualizing viral dynamics, innate immune responses, and host signaling cascades in vivo. Their transparency, genetic tractability, and well‐characterized immune system make them ideal for studying infection biology at single‐cell resolution.^26^, ^27^

**FIGURE 4:**
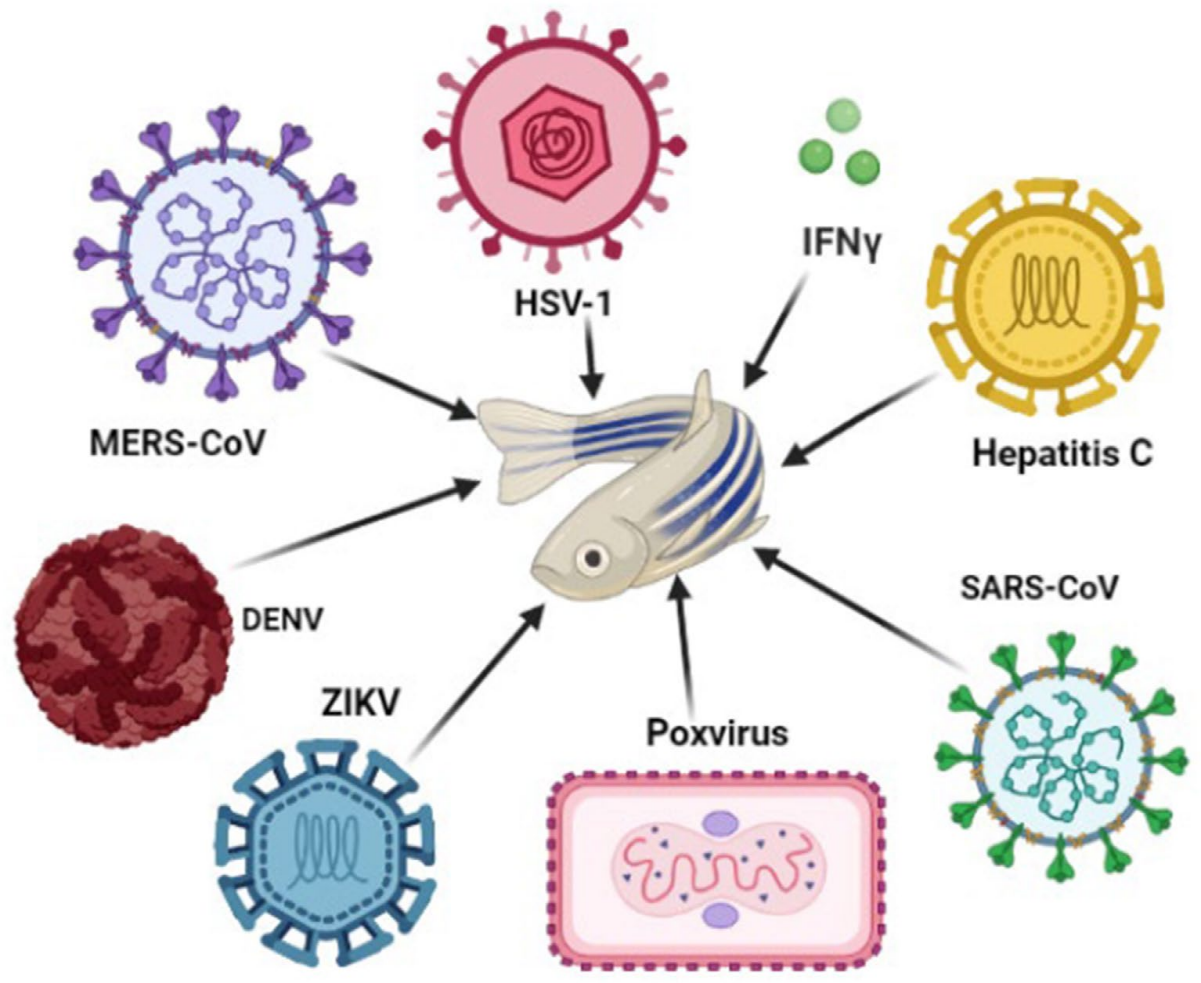
Zebrafish models for studying viral pathogenesis and host–virus interactions. Representative examples include HSV‐1, CHIKV, DENV, HCV, and SARS‐CoV‐2. The figure illustrates infection sites, innate immune responses, and fluorescent visualization of viral replication. Zebrafish are effective for investigating antiviral mechanisms and innate immunity but are not standard productive infection models for several human respiratory viruses; SARS‐CoV‐2 infection in zebrafish is likely limited to abortive replication in the swim bladder.^29^ CHIKV, chikungunya virus; DENV, dengue virus; HCV, hepatitis C virus; HSV, herpes simplex virus; IFN, interferon. Figure created with BioRender.com; minor adjustments made for clarity.

The present review examines how zebrafish can be used to track viral entry, replication, and suppression mechanisms in real time. Moreover, several studies have proposed zebrafish as a platform to explore newly emerging viruses, including members of the Coronaviridae and Poxviridae families^28^ (Figure 4).

### 2.14 Established viral models

Zebrafish, both embryonic and adult, have been utilized to study diverse human viruses and to identify key factors governing viral entry and replication:

- Herpes simplex virus 1 (HSV‐1): Zebrafish embryos have served to identify potential cell‐surface receptors that facilitate HSV‐1 entry and to map infection routes in neural tissues.^30^
- Chikungunya virus (CHIKV): This mosquito‐borne alphavirus, responsible for neurological disease in neonates and severe arthritis in adults, has been shown to replicate efficiently in zebrafish larvae, enabling visualization of viral spread and innate immune activation.^31^
- Dengue virus (DENV): A novel zebrafish model of dengue pathogenesis was developed to evaluate the therapeutic efficacy of the Indian pentaherbal formulation Denguenil Vati. The study demonstrated improvements in cellular and biochemical parameters, highlighting zebrafish as a proof‐of‐concept platform for early drug screening and efficacy testing.^32^
- Hepatitis C virus (HCV): Injection of an HCV subreplicon vector into zebrafish zygotes induced viral core RNA and protein expression and transcriptomic changes similar to human hepatocytes. Treatment with oxymatrine or ribavirin significantly reduced viral RNA and protein levels, demonstrating the model's utility for antiviral compound validation.^33^
- Endogenous and exogenous retroviruses: Zebrafish harbor several endogenous retroelements, providing an opportunity to study retrovirus‐driven genomic alterations and antiviral immunity.^34^

Collectively, these examples establish zebrafish as a versatile system for dissecting host antiviral mechanisms and testing candidate therapies.

### 2.15 Modeling coronaviruses

Middle East respiratory syndrome coronavirus (MERS‐CoV) and severe acute respiratory syndrome coronavirus 2 (SARS‐CoV‐2) are among the most‐studied emerging pathogens causing respiratory illness in humans.^35^, ^36^, ^37^ The SARS‐CoV‐2 spike (S) glycoprotein mediates host cell entry by binding the angiotensin‐converting enzyme 2 (ACE2) receptor and inducing membrane fusion.

Zebrafish possess an ACE2 ortholog, and its expression in developing larvae has prompted investigations into coronavirus tropism. However, current data indicate that zebrafish do not support a productive systemic SARS‐CoV‐2 infection. Instead, abortive viral replication is limited to the swim bladder epithelium, which is evolutionarily related to the mammalian lung.^38^

Despite this, zebrafish provide an excellent platform for studying host immune responses to viral proteins, interferon activation, and cytokine dysregulation. CRISPR/Cas9‐mediated knockouts of immune genes (e.g., *stat1*, *ifnar1*, *irf3*) and transgenic interferon reporter lines have been used to quantify macrophage recruitment, neutrophil dynamics, and antiviral signaling during coronavirus challenge.

### 2.16 Limitations and scope

Although zebrafish are powerful for dissecting antiviral immunity, they are not a universal infection model for all human viruses. Key limitations include the following:

1. Temperature constraint: Zebrafish thrive at 28–29°C, which is below the optimal replication range of many mammalian viruses (35–37°C).
2. Receptor and tropism divergence: Human viral receptors (ACE2, DPP4, CD4, etc.) may differ structurally, resulting in limited binding or abortive infection.
3. Innate‐biased immunity: Larval zebrafish rely mainly on innate immune pathways, whereas adaptive T‐/B‐cell responses develop later, restricting long‐term immune‐memory studies.
4. Experimental infection routes: Microinjection or immersion exposure does not perfectly mimic natural transmission.

Understanding these constraints allows researchers to use zebrafish appropriately for visualizing infection dynamics, host responses, and antiviral mechanisms rather than as a direct substitute for mammalian infection models.^38^

Table 3 *summarizes key experimental recommendations for zebrafish viral disease research, including experimental conditions, host–pathogen interactions, genetic tools, and validation strategies*.

**TABLE 3:** Key experimental recommendations for zebrafish viral disease research.

Aspect	Recommended practice
Focus area	Use for innate immunity, cytokine, and antiviral screening studies, not for full systemic infection
Temperature	Conduct assays at 28–32°C; interpret replication data cautiously
Genetic tools	Use CRISPR/Cas9 knockouts (*stat1*, *ifnar1*, *irf3*) to confirm pathway function
Receptor modeling	Introduce human viral receptors (e.g., human ACE2) via transgenesis for host‐range validation
Translational link	Compare zebrafish results with mammalian infection or cell‐culture models

### 2.17 Additional validated disease areas

Beyond cancer and viral infections, zebrafish have proven highly valuable for modeling a broad spectrum of human diseases, including skeletal, renal, cardiovascular, and hematologic disorders. These models leverage the species' genetic similarity to humans, optical transparency, and rapid development, enabling direct visualization of tissue‐specific pathology and high‐throughput screening of genetic or pharmacological interventions.

Table 4 summarizes representative zebrafish disease models generated through CRISPR/Cas9 or other genome‐editing strategies, highlighting their genetic targets, phenotypic outcomes, and translational significance.

**TABLE 4:** Representative zebrafish–CRISPR/Cas9‐based models for noncancer diseases.

Gene/target pathway	Human disease modeled	Editing strategy	Phenotypic outcome/experimental readout	References
*col1a1a*	Osteogenesis imperfecta	CRISPR knockout	Recapitulates brittle bone phenotype and abnormal collagen deposition	[39]
*sp7 (osterix)*	Osteopetrosis/skeletal dysplasia	Germline knockout	Reduced bone mineralization; used for screening anabolic agents	[40]
*pkd2*	Polycystic kidney disease	CRISPR knockout	Cyst formation in pronephros; validated with rescue using human PKD2 mRNA	[41]
*nphs1 (nephrin)*	Congenital nephrotic syndrome	Morpholino and CRISPR combination	Impaired glomerular filtration barrier; proteinuria phenotype	[42]
*gata1*	Erythropoietic anemia	CRISPR knockout	Decreased erythroid differentiation; restored by human GATA1 mRNA	[43]
*runx1*	Myelodysplastic syndrome	CRISPR knockout	Defective hematopoietic stem cell formation; used for drug rescue assays	[44]
*jak2a*	Myeloproliferative neoplasms	CRISPR knockin (V617F analog)	Recapitulates hyperproliferative hematopoiesis; used for JAK inhibitor screening	[45]

Abbreviation: mRNA, messenger RNA.

Zebrafish have been instrumental in exploring skeletal dysplasias and bone remodeling defects, where mutations in genes such as *col1a1a* and *sp7* lead to phenotypes resembling osteogenesis imperfecta and osteopetrosis. Similarly, renal disease models have been established by targeting genes like *pkd2* and *nephrin (nphs1)*, mimicking polycystic kidney disease and nephrotic syndrome, respectively.

In the hematologic system, zebrafish mutants in *gata1*, *runx1*, and *jak2a* recapitulate anemia, myelodysplasia, and myeloproliferative disorders, offering conserved platforms for drug testing and pathway dissection. Such models underscore the zebrafish's utility for dissecting developmental mechanisms and evaluating small‐molecule therapeutics relevant to human pathophysiology.

Together, these disease models extend the zebrafish's relevance far beyond oncology and virology, solidifying its role as a comprehensive vertebrate system for understanding gene function, disease progression, and therapeutic intervention.

### 2.18 Translational implications

Zebrafish models continue to expand our understanding of gene function across organ systems. Their combination of high fecundity, optical accessibility, and compatibility with CRISPR/Cas9 facilitates the rapid generation of disease‐relevant alleles and in vivo testing of candidate therapeutics. Furthermore, the ability to perform real‐time imaging of tissue damage, regeneration, and immune infiltration distinguishes zebrafish as a uniquely powerful complement to mammalian models. Integration of these approaches across diverse diseases enhances translational insights and accelerates preclinical discovery.

Current challenges and limitations associated with disease modeling in zebrafish.

**FIGURE 5:**
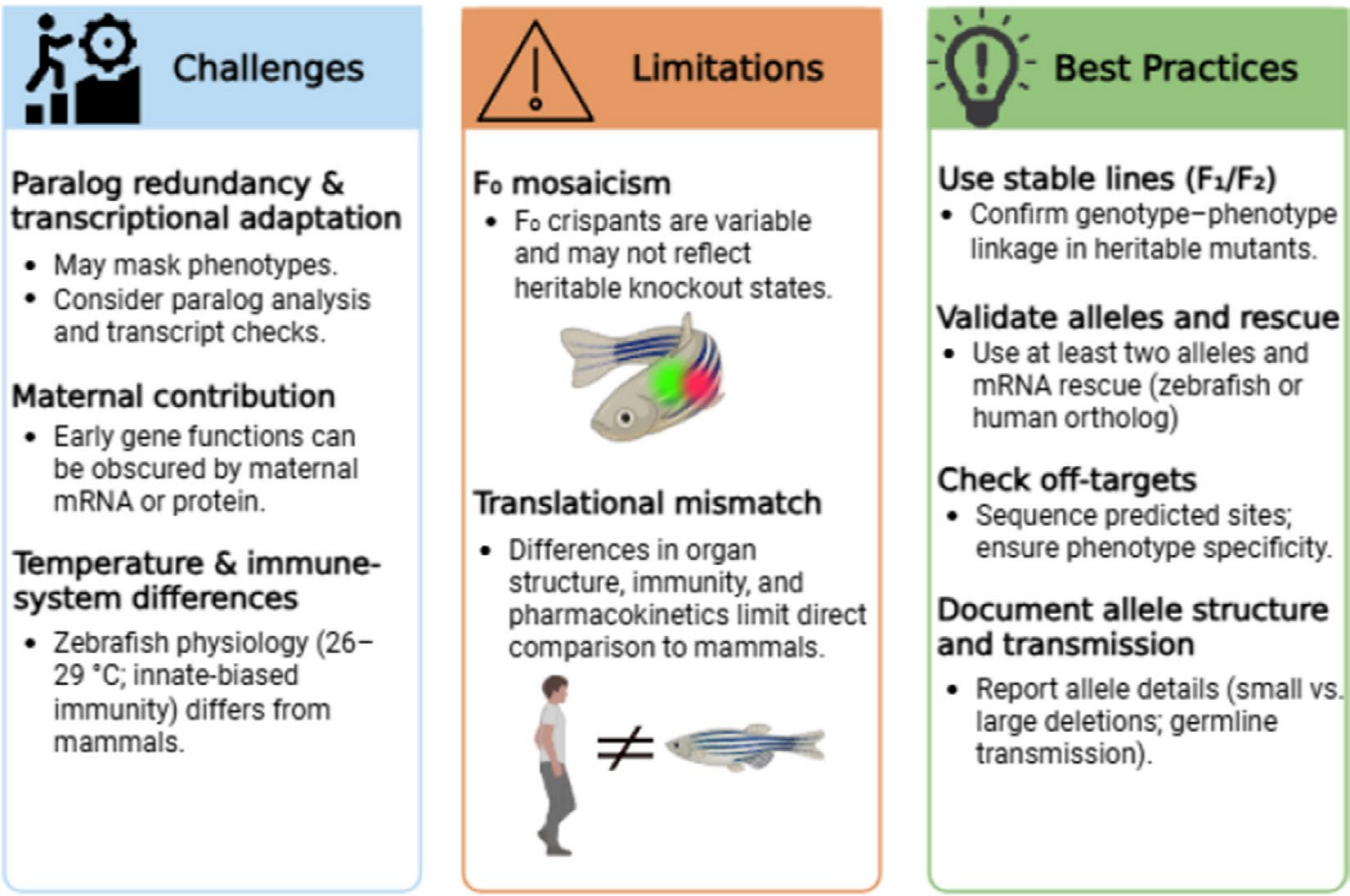
Checklist of current challenges, limitations, and best‐practice recommendations for zebrafish disease modeling. The schematic summarizes major experimental considerations: (1) paralog redundancy and transcriptional adaptation may mask phenotypes; (2) maternal contribution can obscure early gene functions; (3) differences in temperature and immune system can affect translational outcomes; (4) F_0_ mosaic data require confirmation in stable lines; and (5) reproducibility depends on allele validation, rescue, and off‐target assessment. These guidelines promote rigorous use of zebrafish–CRISPR/Cas9 models for human disease research. Figure created with BioRender.com.

Zebrafish models have proven indispensable for exploring vertebrate development and disease mechanisms, yet several biological and methodological constraints must be considered to ensure accurate interpretation and reproducibility. Understanding these limitations is essential for designing rigorous experiments and translating zebrafish findings effectively into mammalian systems (Figure 5).

Some limitations stem from fundamental anatomical and physiological differences between zebrafish and mammals. For instance, zebrafish are ectothermic and maintained at 28–29°C, which can influence metabolic rates, drug pharmacokinetics, and immune responses. Certain mammalian organ analogs—such as lungs, mammary glands, or bone marrow—have only partial counterparts in zebrafish, which may restrict direct modeling of some human conditions.

At the genetic level, teleost‐specific whole‐genome duplication introduces functional redundancy, whereby duplicated genes (ohnologs or paralogs) can compensate for one another. This genetic buffering may mask phenotypes following single‐gene disruption. Additionally, transcriptional adaptation—the compensatory upregulation of related transcripts triggered by mutant mRNA decay—can further obscure loss‐of‐function effects.^3^, ^4^

Another important consideration is the maternal contribution of mRNA and proteins deposited in the oocyte prior to fertilization. These maternal factors can sustain early development even when zygotic gene expression is disrupted, potentially masking essential embryonic functions. When early roles are suspected, researchers should compare maternal, zygotic, and MZ mutants or perform rescue experiments using orthologous mRNAs to verify specificity.^2^

Best practices for rigorous zebrafish disease modeling include the following:

1. Generating at least two independent alleles per target gene: one small frameshift and one larger deletion removing critical domains.
2. Confirming phenotypes through mRNA rescue using either zebrafish or human orthologs.
3. Performing targeted off‐target validation and transparent reporting of allele structures and germline transmission.
4. Accounting for paralog expression and potential compensatory mechanisms when interpreting null phenotypes.

Although these challenges underscore the need for cautious interpretation, they do not diminish the zebrafish's value as a translational model. Instead, addressing them through careful experimental design and transparent validation will enhance the reliability and impact of zebrafish‐based disease research.

## 3 CONCLUSION AND FUTURE PERSPECTIVE

This review highlights how zebrafish (*D. rerio*) have become an indispensable vertebrate model for studying human diseases and for accelerating translational discoveries through CRISPR/Cas9 genome engineering. By combining genetic tractability, optical transparency, and conserved physiology, zebrafish allow researchers to model complex human conditions, including cardiovascular, oncologic, infectious, and metabolic diseases, within a living vertebrate system.

The integration of CRISPR/Cas9 technology has transformed zebrafish research, enabling rapid generation of targeted knockouts, knockins, and precise genetic corrections that faithfully replicate human disease phenotypes. This synergy supports not only gene‐function discovery but also the development and preclinical testing of novel therapeutics.

Although zebrafish models face inherent anatomical and physiological differences from mammals, as discussed in this review, continuous methodological innovation and transparent validation strategies are mitigating these challenges. Rigorous application of best practices, such as verifying phenotypes across independent alleles, using mRNA rescue, and integrating mammalian validation, will ensure that zebrafish remain a reliable and predictive system for translational science.

Looking ahead, the zebrafish–CRISPR/Cas9 platform offers unique opportunities for uncovering the genetic architecture of complex disorders. Emerging research directions include the following:

- Neurodevelopmental and neurodegenerative diseases, where CRISPR/Cas9‐based mutants are already revealing risk genes and pathways implicated in autism spectrum disorder and Alzheimer's disease.
- Ocular malformation syndromes such as microphthalmia, anophthalmia, and coloboma (MAC), where systematic CRISPR/Cas9 mutagenesis and enhancer mapping promise to identify novel regulators of eye development.^46^
- Regenerative medicine and aging research, where zebrafish provide tractable systems for studying tissue repair, stem cell dynamics, and longevity pathways.
- Nutrigenomics and metabolic disorders, where dietary interventions can be tested directly in vivo to assess gene–environment interactions.

In the coming decade, zebrafish are poised to become an even more integral part of functional genomics and precision medicine. Their compatibility with high‐throughput screening, coupled with ethical and economic advantages, positions them as a bridge between molecular discovery and clinical translation.

## AUTHOR CONTRIBUTIONS

**Manikandan Sivaprakasam:** Conceptualization; data curation; formal analysis; investigation; methodology; project administration; resources; software; supervision; validation; visualization; writing – original draft; writing – review and editing. **Aashika Raagavi Jeanpierre:** Formal analysis; validation. **Salma Mohammed:** Formal analysis; validation. **Rajesh Srinivasan:** Formal analysis; validation. **Aman Kumar Mohanty:** Formal analysis.

## FUNDING INFORMATION

This research received no specific grant from any funding agency in the public, commercial, or not‐for‐profit sectors.

## CONFLICT OF INTEREST STATEMENT

The author declares that they have no competing interests.

## ETHICS STATEMENT

None.

## Data Availability

All relevant data and details of resources can be found within the article and its Supporting Information.
